# Calcineurin Participation in Hebbian and Homeostatic Plasticity Associated With Extinction

**DOI:** 10.3389/fncel.2021.685838

**Published:** 2021-06-16

**Authors:** Salma E. Reyes-García, Martha L. Escobar

**Affiliations:** Laboratorio de Neurobiología del Aprendizaje y la Memoria, División de Investigación y Estudios de Posgrado, Facultad de Psicología, Universidad Nacional Autónoma de México, Ciudad de México, Mexico

**Keywords:** extinction, calcineurin, Hebbian and homeostatic plasticity, long-term depression, kinases and phosphatases, depotentiation

## Abstract

In nature, animals need to adapt to constant changes in their environment. Learning and memory are cognitive capabilities that allow this to happen. Extinction, the reduction of a certain behavior or learning previously established, refers to a very particular and interesting type of learning that has been the basis of a series of therapies to diminish non-adaptive behaviors. In recent years, the exploration of the cellular and molecular mechanisms underlying this type of learning has received increasing attention. Hebbian plasticity (the activity-dependent modification of the strength or efficacy of synaptic transmission), and homeostatic plasticity (the homeostatic regulation of plasticity) constitute processes intimately associated with memory formation and maintenance. Particularly, long-term depression (LTD) has been proposed as the underlying mechanism of extinction, while the protein phosphatase calcineurin (CaN) has been widely related to both the extinction process and LTD. In this review, we focus on the available evidence that sustains CaN modulation of LTD and its association with extinction. Beyond the classic view, we also examine the interconnection among extinction, Hebbian and homeostatic plasticity, as well as emergent evidence of the participation of kinases and long-term potentiation (LTP) on extinction learning, highlighting the importance of the balance between kinases and phosphatases in the expression of extinction. Finally, we also integrate data that shows the association between extinction and less-studied phenomena, such as synaptic silencing and engram formation that open new perspectives in the field.

## Introduction

Extinction from the perspective of classical conditioning can be defined as the reduction or temporal inhibition of the conditioned response (CR) that takes place once established a CS-US (conditioned stimulus-unconditioned stimulus) association and the CS is repeatedly presented in the absence of the US, while for instrumental learning, extinction represents the reduction in responses, when a response (R) that was previously followed by an appetitive outcome (O) is not followed by that outcome anymore (Myers and Davis, 2002; Myers et al., 2006; Herry et al., 2010). This reduction of a previously learned behavior is a natural and adaptive process that allows animals to re-learn associations about their environment. Originally, extinction was thought to implicate the elimination of a behavior previously acquired (Rescorla and Wagner, 1972). However, the sudden reappearance of the extinguished behavior (spontaneous recovery), its reappearance by a reminder (reinstatement) or by changing the context (renewal), provided evidence that extinction is not the erasure of certain learning. Instead, extinction refers to a new learning or a re-learning process (Bouton, 2004; Bouton et al., 2011), that also presents acquisition, consolidation, and retrieval phases (Quirk and Mueller, 2008). The mechanisms involving this type of learning are not entirely explored and are yet to be defined.

Behaviorally it has been proposed that during extinction the CS acquires inhibitory properties that suppress the CR (Bouton et al., 2006), or alternatively, that the original CS-US association is modified (Bouton and Todd, 2014). As extinction refers to a new learning, the neural basis of acquisition of conditioning (i.e., synaptic plasticity) have also been associated as underlying mechanisms of extinction, as we will discuss later. Particularly, long-term depression (LTD) has been proposed as the underlying mechanism of extinction, while the protein phosphatase calcineurin (CaN) has been widely related to both the extinction process and LTD.

Calcineurin is a Ca^2+^/calmodulin (CaM)-dependent serine/threonine phosphatase consisting of two subunits: one catalytic (CaN-A, ∼61 kDa) and one regulatory (CaN-B, ∼19 kDa). The mammalian CaN-A has three isoforms: α-isoform is neuron specific, β-isoform has wide distribution and γ-isoform is predominantly located in cortical neurons. Meanwhile, CaN-B has two isoforms: CaN-B1 and CaN-B2, only CaN-B1 binds to CaN-Aα and CaN-Aβ, while CaN-B2 was found only in testes. Each CaN subunit isoform is encoded by an individual gene, which are located in different chromosomes: CaN-Aα in chromosome 4, CaN-Aβ in chromosome 10, and CaN-Aγ in chromosome 8 (Tarasova et al., 2018). In addition to calcineurin, the serine/threonine protein phosphatase family members include protein phosphatases 1 (PP1), 2A (PP2A), and 2C (PP2C) and have different roles on signal transduction in eukaryotic cells (Rusnak and Mertz, 2000). Calcineurin is widely distributed in the body and it is selectively enriched within the post-synaptic densities and cell soma of neurons of the central nervous system (Groth et al., 2003) where primarily modulates synaptic transmission associated with memory (Tarasova et al., 2018). Calcineurin was originally described for its role on the activation of the nuclear factor of activated T-cells (NFAT), this regulation is linked to apoptosis, cardiac pathology and immune response in the kidney (Azzi et al., 2013).

## Mechanisms Underlying Extinction: Hebbian and Homeostatic Plasticity

**FIGURE 1:**
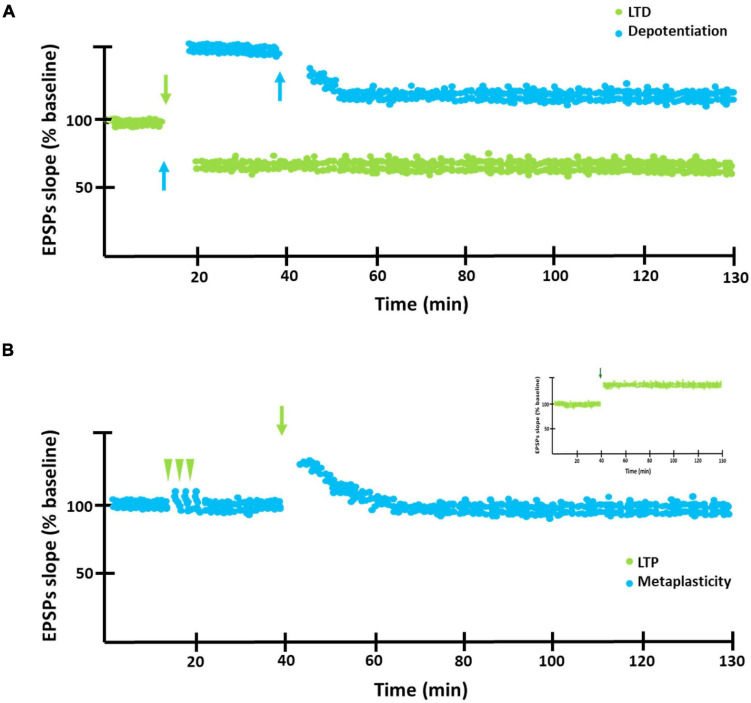
LTD, depotentiation and metaplasticity graphic representation. **(A)** Graphic representation of LTD (green circles) and depotentiation (blue circles). Blue arrows indicate application of low frequency stimulation in order to induce LTD or depotentiation. **(B)** Metaplasticity. After receiving a weak stimulation (green arrow heads), LTP cannot be induced by applying a high frequency stimulation (green arrows) capable of inducing this phenomenon in a control preparation as observed in the inset. That is, the previous stimulation modifies the threshold to induce LTP.

Hebbian synaptic plasticity refers to the activity-dependent modification of the strength or efficacy of synaptic transmission at preexisting synapses and has a central role in the capacity of the brain to incorporate transient experiences into persistent memory traces. In this regard, the synaptic plasticity phenomena known as long-term potentiation (LTP) and long-term depression (LTD), are believed to underlie memory formation and maintenance. LTP refers to the prolonged activity-dependent increment of the synaptic efficacy, usually generated by the application of high frequency stimulation (HFS) and it is associated with the induction and maintenance of conditioning. LTD, in turn, refers to the activity-dependent decrement of the synaptic efficacy, commonly induced by low frequency stimulation (LFS) (Bear et al., 2001; Abraham, 2008; Citri and Malenka, 2008). Similar decrements can be generated after the induction of LTP, in which case the decrease is called depotentiation referring to the reversal of synaptic strength from a potentiated LTP state. In particular, LTD as well as synaptic depotentiation (Figure 1A) have been closely associated with extinction learning (Lin et al., 2003a; Saito et al., 2012; Li et al., 2016).

In this context, the hebbian forms of plasticity would need some type of homeostatic regulation to preserve its adequate function (Turrigiano and Nelson, 2004). Homeostatic forms of plasticity might provide the global regulation necessary to maintain synaptic strength and plasticity within a functional dynamic range. These forms of plasticity also operate through diverse mechanisms: by detecting changes in neuronal firing and scaling excitatory synaptic strengths up or down while preserving their relative weights (synaptic scaling); by strengthening pre- or post-synaptic properties as a compensatory mechanism (synaptic redistribution) or by altering the ability of synapses to undergo subsequent Hebbian modifications (metaplasticity). In this framework, metaplasticity is thought to be essential not only to maintain synapses within a dynamic functional range but also for the maintenance of memory traces (Figure 1B; Abbott and Nelson, 2000; Pérez-Otaño and Ehlers, 2005; Abraham, 2008; Abraham and Richter-Levin, 2018; Lee and Kirkwood, 2019).

Among the evidence supporting the association between LTD and extinction, Dalton and collaborators showed that the inhibition of *N*-Methyl-D-Aspartate receptors (NMDAR)-dependent LTD in the amygdala elicits an impairment of fear extinction training (Dalton et al., 2008), whilst Amano et al. (2010) showed that fear conditioning was associated with an enhancement of field excitatory post-synaptic potentials (fEPSPs) in the basolateral amygdala (BLA), which was partially reverted by the extinction of this task. Similarly, it was reported that the fear conditioning-induced potentiation of EPSCs was reverted by extinction in the thalamus-amygdala and cortico-lateral amygdala pathways (Kim et al., 2007; Hong et al., 2009). Interestingly, Li and collaborators showed that the inhibition of the acid-sensing ion channel 1a (ASIC1a) attenuated the LTD induction in the insular cortex, as well as the extinction of the conditioned taste aversion (CTA) (Li et al., 2016). It was also shown that the application of low-frequency stimulation (LFS) in the hippocampal CA1 area reduced the expression of freezing just like extinction does (Saito et al., 2012). More recently it was observed that optogenetically induced LTD in the thalamus-lateral amygdala (LA) pathway reduced fear conditioning (Lee et al., 2016; Klavir et al., 2017), as well as drug and alcohol-seeking behaviors (Ma et al., 2018; Rich et al., 2019). These findings exemplify the remarkable relationship between LTD and extinction, and strongly suggest that the former represents a mechanism underlying the latter.

There is also a series of studies emphasizing the relationship between the processes of extinction and depotentiation. In this sense, it was proven that several conditioned fear extinction paradigms induce synaptic depotentiation in the lateral amygdala (Lin et al., 2003a; Kim et al., 2007; Hong et al., 2009). Zhang and collaborators, for example, showed that failure in spatial memory extinction is accompanied by impairment in both LTD and synaptic depotentiation (Zhang et al., 2011). Likewise, it was observed that optical depotentiation of auditory pathways to the amygdala generates amnesia of fear conditioning induced by optogenetic stimulation (Nabavi et al., 2014), and optogenetically induced depotentiation of the auditory pathways to LA suppressed conditioned fear responses to the CS (Kim and Cho, 2017). Similarly, Song et al. (2017) described that the administration of the antipsychotic olanzapine in the CA3 area of the hippocampus impaired depotentiation as well as reversal learning in the Morris water maze test.

Taken together, the findings described above clearly show that decrements in synaptic efficacy (either LTD or depotentiation) constitute mechanisms that underlie extinction.

Nowadays, learning and memory research widely accepts that the trace of memory refers to the formation of the engram. In this sense, Lacagnina and collaborators showed that fear extinction suppresses the reactivation of contextual fear engram cells while activating a second ensemble in the hippocampus (Lacagnina et al., 2019). More recently, Zhang and collaborators showed that fear extinction memory requires forming a new engram in the basolateral amygdala (Zhang et al., 2020). Furthermore, chronic stimulation of engram cells associated with fear memories in the hippocampus produced a reduction of fear responses, suggesting that optogenetic manipulation of a fear engram is sufficient to induce an extinction-like behavior (Chen et al., 2019; Cincotta et al., 2021). Likewise, the inhibition of the assembly activated during cue-paired alcohol self-administration in the medial prefrontal cortex (mPFC), 1 month later prevented relapse of alcohol-seeking (Visser et al., 2020), and optogenetic activation of infralimbic cortex-basolateral amygdala pathway during fear extinction trials, resulted in a stronger extinction compared to non-optogenetic activation (Bukalo et al., 2021). From these data, it can be inferred that in fact extinction represents new learning that requires the formation of a new engram. Nevertheless, it has alternatively been proposed that extinction implies the silencing of the original engram. The term silent engram refers to those engrams that cannot be retrieved by natural retrieval cues but can be retrieved with direct optogenetic stimulation (Josselyn and Tonegawa, 2020). Engram silencing also diminishes the previously learned behavior and seems to share synaptic mechanisms with extinction (Josselyn and Tonegawa, 2020). In this sense, it was shown that the chemogenetic reactivation of a fear engram evoked the extinguished behavior (Yoshii et al., 2017). On the other hand, the fact that reconsolidation and extinction seem to share similar signal transduction cascades, including the CaN participation (Fukushima et al., 2014), could imply that memory reconsolidation constitutes a component of extinction. However, these are quite novel ideas that require more experimental support.

So far, we have mentioned evidence showing that LTD and depotentiation are mechanisms underlying extinction. There is also interesting evidence demonstrating the participation of homeostatic plasticity in the regulation of the extinction process. In this sense, it was shown that fear extinction induces LTP instead of LTD in the hippocampal CA1 area, through the activation of mGlu receptors (Stansley et al., 2018), and that CTA extinction impairs LTD induction in the insular cortex (Li et al., 2016). Our group has shown that LTP in the insular cortex promotes CTA retention (Escobar and Bermúdez-Rattoni, 2000). Additionally, we recently showed that the extinction of CTA is bidirectionally modulated by LTP and LTD. While the induction of LTP reinforces the retention of learning, the induction of LTD facilitates its extinction (Rodríguez-Durán et al., 2017). More recently, we also showed that CTA extinction allows the induction but not the maintenance of LTP in the insular cortex *in vivo* (Rivera-Olvera et al., 2018). In like manner, homeostatic plasticity triggered by optogenetic stimulation in the hippocampus altered the balance between excitation and inhibition, thus favoring the extinction expression (Mendez et al., 2018).

Furthermore, it was reported that the odor preference paradigm in rat pups leads to up-regulation of α-amino-3-hydroxy-5-methyl-4-isoxazolepropionic acid (AMPA) and NMDA receptor levels. However, when pups are re-trained 3 h later with another odor, the levels of AMPA and NMDA receptors are reset to the baseline condition through a metaplastic mechanism. This adjustment in receptor levels is associated with down-regulation of the NMDA receptor subunit GluN1 and leads to unlearning of the first odor. In addition, when the phosphatase calcineurin is inhibited in the anterior piriform cortex during training or retraining, the downregulation of GluN1, as well as the unlearning originated by metaplasticity, are prevented (Mukherjee et al., 2017; Bhattacharya et al., 2018). In a similar manner, the negative effect of isoflurane administration in the hippocampus and amygdala on fear memory, was prevented when CaN was inhibited after exposure to isoflurane in these areas (Yang et al., 2017). These results suggest that both calcium levels and calcineurin activity participate in the homeostatic regulation exerted by extinction.

## On the Cellular Basis of Extinction

Derived from the aforementioned evidence, the understanding of the cellular and molecular mechanisms underlying synaptic plasticity expressions becomes essential to comprehend the plastic mechanisms underlying extinction learning.

Concerning the NMDAR-dependent LTP, the binding of glutamate (Glu) and the post-synaptic depolarization allow the calcium ion (Ca^2+^) influx to the postsynaptic membrane. In high concentrations, Ca^2+^ forms a complex with calmodulin, which modifies and activates the protein Ca^2+^/calmodulin-dependent kinase II (CaMKII). CaMKII then phosphorylates the AMPA receptors at residue S831 of the GluA1 subunit, in concert with this, phosphorylation of GluA1 S818 by PKC and S845 by PKA, lead to AMPARs insertion in the post-synaptic membrane (Dunning and During, 2003; Diering and Huganir, 2018). In addition, phosphorylation of GluA1 S831 by CaMKII or PKC regulates the conductance of AMPA receptors (Roche et al., 1996; Barria et al., 1997; Mammen et al., 1997; Jenkins and Traynelis, 2012; Coultrap et al., 2014; Diering et al., 2016). Moreover, phosphorylation of auxiliary transmembrane AMPA receptor regulatory proteins (TARPs) subunits by CaMKII and PKC play prominent roles in regulating AMPAR synaptic localization during LTP and LTD (Rouach et al., 2005; Tomita et al., 2005; Menuz et al., 2008; Kristensen et al., 2011; Jenkins and Traynelis, 2012; Park et al., 2016). However, it has been described that CaMKII can also be activated by LFS, but in this case its activity elicits the phosphorylation of the GluA1-S567, thus promoting the removal of AMPA receptors toward extrasynaptic sites, which in turn promotes the induction of LTD (Coultrap et al., 2014; Woolfrey et al., 2018). On the other hand, low levels of Ca^2+^ activate calcineurin (CaN), a serine/threonine protein phosphatase, which dephosphorylates the inhibitor 1 (I-1) that normally acts as an inhibitor of the protein phosphatase (PP1). When I-1 is dephosphorylated by CaN, the now active PP1 dephosphorylates CaMKII thereby causing its inhibition. Additionally, CaN can dephosphorylate GluA1 S845 and TARPs to control LTD (Tavalin et al., 2002; Tomita et al., 2005; Diering et al., 2014; Itakura et al., 2014). This generates the removal of AMPA receptors from the membrane and its internalization, which leads to a decrement of the synaptic efficiency that can generate LTD.

The mechanisms that generate NMDAR-dependent LTD are also involved in the induction of depotentiation, and are generally triggered by LFS, but can also be activated by spike timing-dependent plasticity (STDP) protocols, a phenomenon in which the precise timing of spikes affects the direction and magnitude of changes in synaptic strength. Typically, a pre-synaptic spike preceding a post-synaptic spike within a narrow time window leads to LTP, if the order is reversed, LTD results (Wittenberg and Wang, 2006; Shouval et al., 2010).

Long-term depression also exists in a metabotropic receptor (mGluR) dependent variant, which can be induced by paired-pulse LFS, or by the application of mGluR agonists such as 1-amino-1,3-dicarboxycyclopentane (ACPD) or (S)-3,5-dihydroxyphenylglycine (DHPG) (Collingridge et al., 2010). DHPG is commonly used for characterizing the induction and expression mechanisms underlying hippocampal mGluR-LTD. The mGluR-LTD has different mechanisms from NMDAR-LTD, but also leads to AMPAR internalization (Gladding et al., 2009). The stimulation of group 1 mGluRs leads to activation of the phosphoinositide-specific phospholipase C (PLC), which allows the activation of the protein kinase C (PKC); PKC can then phosphorylate the AMPA receptors thus allowing their removal from the synaptic space. The canonical signaling pathway of group I mGluRs involves the hydrolysis of phosphatidyl inositol to generate inositol trisphosphate (IP3) and diacylglycerol (DAG), which in turn can activate PKC. This pathway is involved in mGluR-LTD triggered by both mGlu1 and mGlu5 receptors in the hippocampus. The protein interacting with C kinase 1 (PICK1) is also required for mGluR-LTD at different synapses. In the perirhinal cortex PICK1 forms a complex with the prototypic member of the neuronal calcium sensor (NCS) family NCS-1, which could act as a high-affinity Ca^2+^ sensor for mGluR-LTD. As PICK1 can also bind PKCα, it is possible that NCS-1 attracts the PICK1–PKC complex to the GluA2 subunit of AMPARs in response to Ca^2+^ signals, resulting in phosphorylation of the subunit and dissociation from the AMPAR-binding protein–glutamate receptor interacting protein (ABP–GRIP) (Lin and Huganir, 2007; Collingridge et al., 2010). Depending on the developmental stage of the animal, mGluR-LTD can be induced in presence of protein synthesis inhibitors (Huber et al., 2000; Nosyreva and Huber, 2005). Other molecular actors that have been implicated in the expression of mGluR-LTD are the p38 mitogen-activated protein kinase (p38 MAPK) and, to a lesser extent, extracellular signal-regulated kinases (ERKs), protein tyrosine phosphatases (PTPs) and Arc, though, the downstream effectors have not been fully described (Collingridge et al., 2010).

Other forms of LTD have also been described, such as endocannabinoid-LTD (eCB-LTD) which is the form of long-term reduction of the neurotransmitter release at the same or nearby synapses by activation of presynaptic cannabinoid receptor type 1 (CB1R) (Xu and Chen, 2015). Induction of eCB-LTD requires an increase in intracellular Ca^2+^ and activation of postsynaptic mGluRs in most brain regions (Xu and Chen, 2015). In the hippocampus, the endocannabinoids act as mediators of a form of heterosynaptic mGluR induced LTD. Endocannabinoid release during LTP can also lead to LTD of GABA (γ-aminobutyric acid)-mediated synaptic transmission and this affects the subsequent plasticity of the network (Collingridge et al., 2010; Fontaine et al., 2020). Endocannabinoids can also function as retrograde messengers in the striatum, neocortex and cerebellum (Collingridge et al., 2010). It is known that activation of CB1R on GABAergic nerve terminals inhibits adenylcyclase, decreases cAMP levels, reduces PKA activity, and increases intraterminal Ca^2+^ thus activating calcineurin. Together, these actions promote dephosphorylation of proteins necessary for transmitter release, leading to inhibitory LTD of transmission (Bennett et al., 2017). However, the exact mechanism of sustaining the long-term depression of a neurotransmitter release after activation of CB1R within the short-time scale (minutes) is still unknown, but the distribution of CB1R largely determines the strength of eCB-mediated short and long-term synaptic plasticity (Xu and Chen, 2015). The activation of CB1R can also be modulated by glucocorticoids and BDNF, which could associate eCB-LTD with different types of learning, including reward-seeking behavior and fear learning (Bennett et al., 2017; Bilbao et al., 2020; Gunduz-Cinar, 2021). Though CB1R activity seem to be also associated to extinction, maybe through the endogenous release of BDNF (Bennett et al., 2017). Nevertheless, NMDAR-LTD has been further explored and predominantly associated with extinction learning (Huang et al., 2016).

## The Molecular Balance: On Calcineurin, Kinases and Other Phosphatases

At present, it is of common knowledge in the field that the activity of kinases, such as CaMKII, has been identified as a molecular mechanism underlying LTP, while the activity of phosphatases, such as CaN, has been associated with LTD expression. Although, as we will discuss later, this perspective begins to be nuanced by experimental evidence.

### Calcineurin Modulation of Learning: Hebbian and Homeostatic Plasticity

**FIGURE 2:**
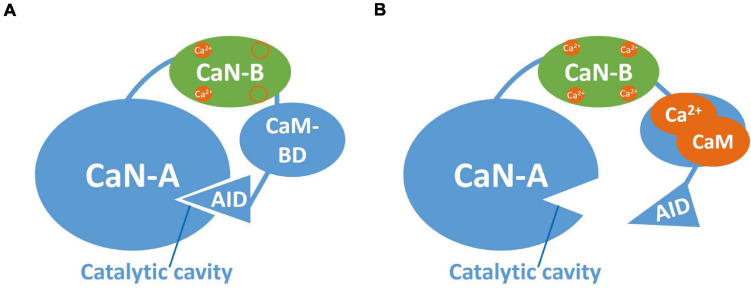
Calcineurin structure. **(A)** Inactivated CaN. Representation of CaN-A catalytic subunit (blue) and the regulatory subunit CaN-B (green). CaN-A consists of a catalytic cavity, a CaN-B binding domain, a calmodulin binding domain (CaM-BD) and an autoinhibitory domain (AID). CaN-B possesses four binding sites for Ca^2+^, two of high affinity typically occupied by Ca^2+^ at basal levels. The AID occupies the catalytic cavity when CaN is inactive. **(B)** Activated CaN. At low Ca^2+^ influx, Ca^2+^ binds to the low affinity sites at CaN-B and the Ca^2+^/CaM complex binds to CaM-BD allowing AID removal from the catalytic cavity.

The evaluation of the protein phosphatases’ participation in extinction has provided valuable information about the cellular mechanisms that underlie this process. Notably, the protein phosphatase 2B (PP2B) or calcineurin plays an essential role in a variety of biological processes, including extinction and LTD, as mentioned earlier. Calcineurin is a serine/threonine phosphatase consisting of two subunits: a catalytic subunit (CaN-A) and a regulatory subunit (CaN-B). The catalytic subunit, in turn, consists of the catalytic region, one binding domain for CaN-B, a binding domain for calmodulin and an autoinhibitory domain (AID). On the other hand, the regulatory CaN-B subunit possesses four domains for calcium, two with low and two with high affinity (Figure 2A; Rusnak and Mertz, 2000; Tarasova et al., 2018).

Regarding the participation of CaN during the extinction process, it was shown that the administration of two selective inhibitors of CaN, cyclosporine A (CsA) or tacrolimus (FK506) before extinction training, blocked fear extinction (Lin et al., 2003b; Fuente et al., 2014; Almeida-Correâ et al., 2015). Shaw and collaborators showed that CaN also plays an important role in the extinction of spatial memories (Shaw et al., 2012). We have recently reported that after CTA extinction, the calcineurin expression is increased in the insular cortex (Rivera-Olvera et al., 2018). Similarly, increases of CaN-A were observed after fear extinction (Alvarez-Ricartes et al., 2018). Furthermore, a recent study shows that the overactivation of CaN, by the administration of chlorogenic acid (CGA) after extinction training, facilitates the extinction and protects from the reinstatement of a cocaine cue memory; this effect was blocked by FK506 (Rich et al., 2020).

The effects of CaN inhibition seem to be phylogenetically conserved, as the administration of protein phosphatases inhibitors blocked the spike frequency decline observed after an *in vitro* extinction procedure of classical conditioning in *Hermissenda crassicornis* (Cavallo et al., 2014).

Calcineurin was also reported to have a role in synaptic plasticity. The administration of cyclosporine or FK506 in a slice preparation of the CA1 area of the hippocampus showed a diminished LTD compared with controls (Mulkey et al., 1994). In a similar preparation, mice lacking the regulatory subunit of CaN also showed a diminished LTD (Zeng et al., 2001). Moreover, the infusion of calcineurin inhibitors blocked the induction of depotentiation in the lateral nucleus of the amygdala (Lin et al., 2003a).

The manipulation of the endogenous regulators that modulate CaN activity has also stressed the role of this phosphatase in synaptic plasticity. In this sense, Zhu and collaborators showed that the knock-out of calpain-1 (a protease that degrades the regulatory subunit of CaN) impaired hippocampal mGluR-LTD, as well as fear extinction (Zhu et al., 2017). Furthermore, the inhibition of calpain before or after extinction training impaired the extinction of fear memory in mice (Song et al., 2018). In addition, the phosphorylation of the endogenous regulator of CaN (RCAN1) by glycogen synthase kinase 3 beta (GSK3β) was proven to be necessary for LTD expression in CA1 synapses, while the phosphorylation of RCAN1 at PKA sites blocked CaN activity, thus allowing the induction of LTP (Dudilot et al., 2020).

There is experimental evidence showing that CaN is also capable of modulating LTP. Funauchi and collaborators showed that CaN inhibition facilitated the induction of LTP in the rat visual cortex (Funauchi et al., 1994). On the other hand, Moradpour and collaborators showed that the inhibition of CaN prevents impairment of hippocampal CA1 LTP by the steroid nandrolone (Moradpour et al., 2019). It is important to mention that it was demonstrated that LTP at the parallel fiber (PF)-Purkinje cell synapses, requires the activation of calcineurin as well as other phosphatases (Belmeguenai and Hansel, 2005; Jörntell and Hansel, 2006; Grasselli and Hansel, 2014). In addition, Fuji and Hirano showed that a late phase of LTD at Purkinje neurons requires a downregulation of calcineurin (Fuji and Hirano, 2002). There is also some behavioral evidence that CaN is involved in the formation of learning since the chronic administration of the CaN inhibitor CsA impaired visuospatial learning (Speigel et al., 2019). It was also recently shown that the maintenance of the long-term memory in an object recognition paradigm is regulated in part by CaN, since the daily systemic administration of FK506 showed extended memory on the task (Sachser et al., 2016).

Dephosphorylation of the AMPAR subunit GluA1 by CaN seems to be a molecular event that may modulate both hebbian and homeostatic plasticity. It was reported that cultured cortical neurons in the presence of tetrodotoxin (TTX) presented reduced CaN activity and high levels of phosphorylated GluA1, associated with increases in the average mEPSC amplitude, thus suggesting that the decreased activity of CaN lead to increased phosphorylation of GluA1, allowing the expression of homeostatic plasticity and favoring in turn the expression of LTP (Kim and Ziff, 2014). Furthermore, it was shown that the balance of AKAP5 (AKAP79/150)-anchored PKA and CaN signaling regulate GluA1 S845 phosphorylation to control homeostatic synaptic plasticity in both hippocampal and cortical neurons (Diering et al., 2014; Sanderson et al., 2018). Indeed, the inhibition of CaN by FK506 leads to an increase in the levels of GluA1 in the olfactory bulb only after an odor preference learning (Bhattacharya et al., 2018). These findings show that CaN is a key molecule not only for LTD modulation, but also for LTP and homeostatic plasticity modulation.

### PP2A Modulation of Synaptic Plasticity and Learning

Similarly, other phosphatases have also been implicated on synaptic plasticity. The protein phosphatase 2A (PP2A) belongs to the serine/threonine protein phosphatase family and have some roles in synaptic plasticity and learning as well. Transgenic mice expressing Simian Virus 40 small-t antigen, which inhibits the PP2A, exhibited a blockade of NMDAR-LTD, as well as deficits in behavioral flexibility (Nicholls et al., 2008). More recently, the intraventricular administration of okadaic acid (OKA), an inhibitor of the protein phosphatase family, attenuated the fEPSP slope and population spike (PS) amplitude of hippocampal dentate gyrus neurons following paired-pulse and HFS (Hamidi et al., 2019). In addition, as mentioned earlier LTP at the PF-Purkinje cell synapses, requires the activation of protein phosphatases as PP1, PP2A, and calcineurin (Belmeguenai and Hansel, 2005; Jörntell and Hansel, 2006; Grasselli and Hansel, 2014). In this regard, several studies have demonstrated that LTD induction requires, or is supported by, the suppression of phosphatases (Ajima and Ito, 1995; Eto et al., 2002; Launey et al., 2004; Kawaguchi and Hirano, 2013). In a recent study, a knock-out for PP2A impaired contextual fear memory extinction, as well as LTD. Surprisingly, the knock-out also disrupted HFS-induced LTP (Wang et al., 2019). Conversely, administration of LB100, a PP2A inhibitor, during conditioning promoted the extinction of methamphetamine induced conditioned place preference in mice (Qian et al., 2020). This evidence suggests that phosphatases of this family may have a broader role in plasticity and learning, however, more experiments are needed to elucidate their participation and possible mechanisms.

### LTD and Extinction Modulation by Kinases: CaMKII, AMPK, MET Receptor, and ERK

Growing experimental evidence shows that kinases also play an important role in LTD and extinction. As we mentioned earlier, CaMKII phosphorylates the GluA1-S567 on AMPARs to promote LTD expression (Coultrap et al., 2014; Woolfrey et al., 2018). In addition, the expression of cerebellar LTD depends on the activity of kinases such as PKA (Grasselli and Hansel, 2014). A recent study showed that overactivation of adenosine monophosphate-activated protein kinase (AMPK) before extinction training in the hippocampus promotes fear extinction maintenance (Wang et al., 2020). Moreover, a knock-out for the MET receptor tyrosine kinase exhibited increases in both LTP and LTD in the hippocampus, as well as faster fear-conditioning learning and conditioned fear-extinction (Xia et al., 2021). It also was recently suggested that extracellular-regulated protein kinase (ERK) activity is important for the regulation of extinction learning expression. In this sense, the inhibition of ERK before the extinction training in the amygdala, hippocampus, medial pre-frontal cortex and nucleus accumbens, facilitated extinction and prevented reconsolidation and reinstatement in both inhibitory avoidance and conditioned place preference tasks (Fukushima et al., 2021; Qiao et al., 2021). Taken this evidence, it seems that phosphatases’ and kinases differential role in learning and memory processes should be reconsidered.

## Calcineurin as a Regulator of Extinction: Molecular Mechanisms on Behavior, Hebbian and Homeostatic Plasticity

As the activity of phosphatases seems to be relevant to extinction learning, knowing their mechanisms of action could lead to a better understanding of the extinction process. For this, we will now focus on the activation and inactivation mechanisms of CaN.

### Activation and Inhibition of CaN

**FIGURE 3:**
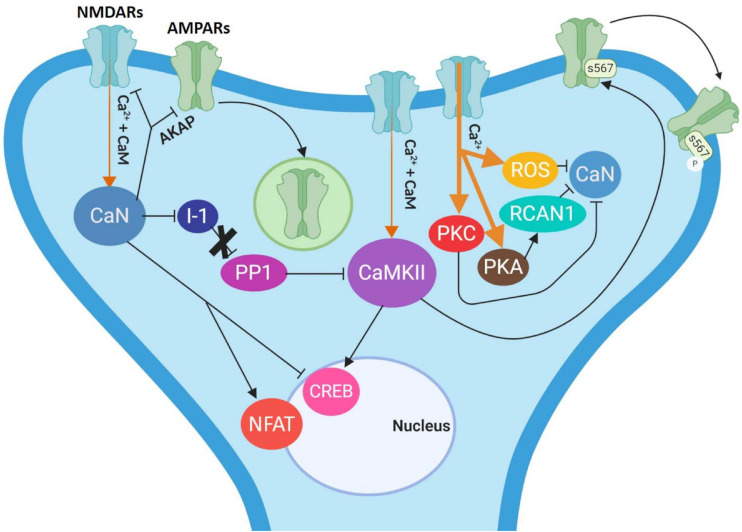
Molecular modulation of extinction. In low concentrations (thin orange arrows), the Ca^2+^/calmodulin complex activates CaN, which then dephosphorylates the I-1 (inhibitor 1), allowing PP1 to dephosphorylate CaMKII. CaN also dephosphorylates NMDARs (in blue) and AMPARs (in green) through AKAPs leading to AMPARs endocytosis, thus promoting LTD induction. NMDAR stimulation that induce LTD may also activate CaMKII, which elicits the phosphorylation of the AMPARs at residue S567 of the GluA1 subunit, propitiating their removal to the extra-synaptic space. CaN dephosphorylates CREB and the NFAT transcription factors as well. High concentrations of Ca^2+^ (thick orange arrows) activate kinases such as PKC and PKA, ROS and RCAN1 may act as CaN endogenous inhibitors. Arrows indicate activation while T arrows indicate inhibition either by phosphorylation or dephosphorylation. I-1: inhibitor 1; PP1: protein phosphatase 1; NFAT: nuclear factor of activated T-cells; CREB: cAMP response element binding; AKAP: A-Kinase Anchoring Protein; ROS: reactive oxygen species; PKA: protein kinase A; PKC: protein kinase C.

In the inactive state of CaN, the AID is located in the catalytic region of CaN-A. As we described before, CaN is activated when there is a low influx of Ca^2+^ (1 μM), which causes Ca^2+^ to bind simultaneously to calmodulin and CaN-B. In turn, the Ca^2+^-calmodulin complex binds to its domain in CaN-A, promoting the removal of the AID from the catalytic region, thus generating the active form of CaN (Rusnak and Mertz, 2000; Figure 2B). Meanwhile, as described above, the active form of CaN dephosphorylates the inhibitor of PP1. Once this happens, the PP1 can dephosphorylate CaMKII, leading to its inhibition. CaN is able to dephosphorylate the AMPA receptors, attaching them through A-kinase anchor proteins (AKAPs), leading to AMPA receptors internalization (Sanderson et al., 2016, 2012). CaN is also capable of directly dephosphorylating NMDAR as well as some transcription factors (e.g., CREB and NFAT), thus altering gene expression (Figure 3; Dunning and During, 2003; Groth et al., 2003; Tarasova et al., 2018). All these processes are associated with the induction of LTD, as we mentioned before.

In contrast, the endogenous inhibition of CaN has not been completely understood. Nevertheless, it is known that it occurs at a high intracellular Ca^2+^ concentration (5 μM). It has been described, *in vitro*, that high concentrations of Ca^2+^ activate kinases, such as CaMKII and PKC, which may phosphorylate and inhibit CaN directly (Hashimoto and Soderling, 1989; Martensen et al., 1989). PKA can be activated as well and then phosphorylate the regulating protein of CaN (RCAN1), thus inhibiting this phosphatase (Kim et al., 2015). The high Ca^2+^ concentrations can also activate reactive oxygen species (ROS), which can bind to the zinc region of the CaN catalytic domain leading to its inhibition (Wang et al., 1996). Conversely, Rusnak and Reiter described that CaN directly modulates ROS (Rusnak and Reiter, 2000).

Besides this modulation by Ca^2+^ intracellular levels, endogenous inhibitors of CaN have been described, such as Cain and Cabin (Lai et al., 1998; Sun et al., 1998), as well as CaN-B homologous peptides (Lin et al., 1999; Parry and June, 2003). Cain is a soluble cytosolic protein presenting a pattern of expression in the brain that closely resembles that of calcineurin. Cain binds to both CaN-A and CaN-B, inhibiting calcineurin and suppressing synaptic vesicle endocytosis (Lai et al., 2000, 1998). Cabin 1, on the other hand, is a nuclear protein that requires an hyperphosphorylated state dependent on both calcium signals and PKC activation, to produce a high-affinity interaction with CaN (Sun et al., 1998).

Alternatively, inhibition of CaN can be generated by synthetic inhibitors such as CsA and FK506. Both are immunosuppressants and bind to immunophilins forming complexes, cyclophilin with CsA and the binding protein of FK506 (FKBP12) in the case of FK506. These two complexes inhibit CaN in a very similar fashion, binding the region between CaN-B and CaN-A subunits, eliciting the split of the Ca^2+^/calmodulin complex. This allows the AID to bind with the catalytic region, once more producing a state of inhibited CaN.

### CaN as a Modulator of Receptor Mobility: NMDA and AMPA Receptors Participation in Extinction

We will now focus on the experimental evidence, highlighting the participation of NMDA and AMPA receptors in the extinction process. Hence, the role of CaN as a regulator of the mobility of these receptors, through the mechanisms described above, becomes particularly important during the expression of extinction.

#### NMDA Receptors

In this sense, the administration of AP5 (an antagonist of the NMDAR) into the amygdala during extinction training blocked the extinction of conditioned fear (Falls et al., 1992). Moreover, the administration of AP5 into the lateral amygdala (LA) also blocked the induction of LTD, preventing the endocytosis of AMPA receptors (Yu et al., 2010). Furthermore, the administration of CPP, another antagonist of NMDAR, during extinction training allowed the acquisition of fear conditioning extinction but blocked its retrieval, which allows to conclude that the consolidation of extinction requires NMDARs (Santini et al., 2001). Sotres-Bayón and collaborators reported that the selective blockade of the NMDARs subunit GluN2B with ifenprodil before extinction training impaired the acquisition and retrieval of fear extinction (Sotres-Bayon et al., 2007). Additionally, it was shown that GluN2A and GluN2B play differential roles in the acquisition and extinction of conditioned fear. The blockade, of GluN2A by NVP-AAM077 before conditioning, impairs acquisition but not extinction, and the blockade of GluN2B, by Ro25-6981, disrupts extinction but not acquisition. These results show a differential role of the NMDARs conformation on conditioning and extinction learning (Dalton et al., 2012). Moreover, the blockade of NMDAR and the inhibition of PKA, CaMKII and MAPK before or after the first test session in a fear-potentiated startle paradigm impaired the extinction, meaning that these molecules must be part of the underlying mechanisms of extinction learning (Szapiro et al., 2003).

Following this line of ideas, the administration of D-cycloserine (DCS), a partial agonist of the NMDARs, has been reported to facilitate fear extinction (Walker et al., 2002; Ledgerwood et al., 2003, 2005; Woods and Bouton, 2006; Bouton et al., 2008). In addition, DCS administration during extinction training promoted the internalization of the AMPA receptors subunit GluA1 as well as fear-extinction learning (Mao et al., 2008).

More recently, it was shown that D-serine, the endogenous co-agonist of NMDARs, is required for the extinction of cocaine-induced behavioral sensitization and for the establishment of LTD (Liu et al., 2016).

#### AMPA Receptors

On the other hand, the systemic administration of the AMPA receptor agonist, PEPA {4-[2-(phenylsulfonylamino) ethylthio]-2,6-difluorophenoxyacetamide}, before extinction training led to facilitation of contextual fear extinction. This effect was blocked by the administration of NBQX, an AMPA receptor antagonist (Zushida et al., 2007). Additionally, AMPARs endocytosis blockade during the initial extinction session disrupts both the expression and recall of fear extinction (Dalton et al., 2008). In a series of studies Clementine and Huganir showed that PKA phosphorylation of GluA1 S845 promotes synaptic insertion of GluA1 Ca^2+^-permeable AMPARs at synapses in the amygdala during fear conditioning to prime subsequent extinction that removes AMPA receptors *via* LTD or depotentiation (Clementine and Huganir, 2010, 2013). Correspondingly, the blockade of GluA2/AMPAR removal in the hippocampus during conditioning prevented the decay of long-term object location memories and impaired depotentiation but not induction of LTP (Migues et al., 2016). In addition, the administration of naltrexone (an antagonist of the μ-opioid receptors) before acquisition promoted AMPAR phosphorylation and its consequent insertion into the membrane, thus protecting memory from extinction (Kibaly et al., 2016).

As we have described, AMPAR trafficking is important for the expression of Hebbian plasticity, but it is also a fundamental process in homeostatic plasticity. For instance, it has been shown that the GluA2 subunit dephosphorylation for both Hebbian and homeostatic plasticity leads to AMPARs internalization, promoting mGluR-LTD and homeostatic synaptic downscaling (Gladding et al., 2009; Cingolani et al., 2019).

These processes occur through different induction mechanisms in the case of mGluR-LTD, mGluR1/5 (group I metabotropic glutamate receptors) are activated by synaptically released glutamate, whereas in the case of homeostatic synaptic downscaling, mGluR1/5 are activated by the immediate early gene Homer1a. The mGluR1/5 signaling then regulates HCN (hyperpolarization-activated, cyclic nucleotide-gated) channel activity, which is also closely involved in the homeostatic plasticity regulation (Cingolani et al., 2019). These mechanisms are still barely explored in relation to extinction. As an example, a recent study showed that mRNA expression of Homer1a in the hippocampus increased after fear extinction (Clifton et al., 2017).

### Metaplastic Modulation of Extinction by Kinases/Phosphatases and Neurotrophic Factors

To finish, it is relevant to mention that metaplasticity differential regulation by kinases/phosphatases or neurotrophic factors could also play a role on extinction, e.g., our group reported that the blockade of PKC but not PKA prevented the LTP impairment produced by CTA training (Rodríguez-Durán and Escobar, 2014), thus revealing differential roles of protein kinases on metaplasticity. On the other hand, we have also reported that the infusion of brain-derived neurotrophic factor (BDNF) in the insular cortex promotes CTA-extinction (Rodríguez-Serrano et al., 2014), showing that BDNF is a key regulator and mediator in the extinction process. This metaplastic view of extinction could guide research toward new perspectives that include other processes, factors and modulators potentially involved in extinction.

## Discussion

We have described experimental evidence supporting that low Ca^2+^ influx triggers the activation of CaN, which in turn leads to the AMPARs internalization, CaMKII inactivation and CREB repression, thus promoting the expression of LTD, depotentiation and extinction.

Nowadays, the notion still prevails that while kinases preferentially participate in the conditioning process, phosphatases underlie extinction (Pagani and Merlo, 2019). Nevertheless, recent evidence begins to present a nuanced approach of such concepts, since it has been shown that phosphatases are involved in the generation of LTP, while kinases are also involved in the expression of LTD (Coultrap et al., 2014; Sanderson et al., 2016; Woolfrey et al., 2018; Wang et al., 2019; Purkey and Dell’Acqua, 2020; Xia et al., 2021). Likewise, there is some evidence associating LTD to conditioning (Altinbilek and Manahan-Vaughan, 2009; Bilbao et al., 2020; Haley et al., 2020) and the activity of kinases to extinction (Wang et al., 2020; Fukushima et al., 2021; Qiao et al., 2021; Xia et al., 2021). Because of that, synaptic plasticity as a general phenomenon underlying learning and memory might be a more suitable concept than LTP and LTD as the respective underlying mechanisms for conditioning and extinction.

These recent data suggest that the molecular actors of conditioning and extinction may not be dissociated nor distinct. Instead, the same molecular elements could be contributing to both processes, with different targets, probably depending on different cellular conditions (e.g., levels of Ca^2+^), which must be considered for further explorations.

Furthermore, homeostatic plasticity should also be considered for further research on the extinction memory field. Indeed, we have described some shared mechanisms between Hebbian and homeostatic plasticity that allow similar outcomes, such as AMPARs internalization, depotentiation or LTD. We have also presented evidence that extinction could lead to metaplastic changes (Rivera-Olvera et al., 2018). With this full view, however, we may also question whether extinction could be considered as a behavioral metaplastic process itself. Although there is evidence that extinction requires the formation of a new engram (Zhang et al., 2020), there is also evidence that extinction could be a synaptic silencing mechanism (Arendt et al., 2013). These findings open new perspectives in the field. In this sense, we should contemplate the participation of homeostatic plasticity, synaptic silencing, as well as the balance between phosphatases and kinases for a broader study of extinction learning.

## Author Contributions

SR-G: conceptualization, drafting the article, and revising it critically for important intellectual content. ME: conceptualization and design, drafting the article and revising it critically for important intellectual content, and funding acquisition. Both authors contributed to the article and approved the submitted version.

## Conflict of Interest

The authors declare that the research was conducted in the absence of any commercial or financial relationships that could be construed as a potential conflict of interest.
